# Inter-district and Wealth-related Inequalities in Maternal and Child Health Service Coverage and Child Mortality within Addis Ababa City

**DOI:** 10.1007/s11524-024-00836-0

**Published:** 2024-03-27

**Authors:** Wubegzier Mekonnen, Worku Dechassa, Desalegn Y. Melesse, Natalia Tejedor-Garavito, Kristine Nilsen, Theodros Getachew, Shegaw Mulu, Naod Wondrad

**Affiliations:** 1 School of Public Health, College of Health Sciences, Addis Ababa University, Addis Ababa, Ethiopia; 2 Institute for Global Public Health, Department of Community Health Sciences, University of Manitoba, Winnipeg, Canada; 3 World Pop Research Group, School of Geography and Environmental Science, University of Southampton, Southampton, UK; 4 Department of Social Statistics and Demography, University of Southampton, Southampton, UK; 5 Health System & Reproductive Health Research Directorate, Ethiopian Public Health Institute, Addis Ababa, Ethiopia; 6 Planning, Monitoring and Evaluation Directorate, Ministry of Health, Addis Ababa, Ethiopia

**Keywords:** Maternal and child health service coverage, Child mortality trends, Spatial analysis, Urban inequalities, Addis Ababa

## Abstract

In sub-Saharan Africa, urban areas generally have better access to and use of maternal, newborn, and child health (MNCH) services than rural areas, but previous research indicates that there are significant intra-urban disparities. This study aims to investigate temporal trends and geographic differences in maternal, newborn, and child health service utilization between Addis Ababa’s poorest and richest districts and households. A World Bank district-based poverty index was used to classify districts into the top 60% (non-poor) and bottom 40% (poor), and wealth index data from the Ethiopian Demographic and Health Survey (EDHS) was used to classify households into the top 60% (non-poor) and bottom 40% (poor). Essential maternal, newborn, and child health service coverage was estimated from routine health facility data for 2019–2021, and five rounds of the EDHS (2000–2019) were used to estimate child mortality. The results showed that service coverage was substantially higher in the top 60% than in the bottom 40% of districts. Coverage of four antenatal care visits, skill birth attendance, and postnatal care all exceeded 90% in the non-poor districts but only ranged from 54 to 67% in the poor districts. Inter-district inequalities were less pronounced for childhood vaccinations, with over 90% coverage levels across all districts. Inter-district inequalities in mortality rates were considerable. The neonatal mortality rate was nearly twice as high in the bottom 40% of households' as in the top 60% of households. Similarly, the under-5 mortality rate was three times higher in the bottom 40% compared to the top 60% of households. The substantial inequalities in MNCH service utilization and child mortality in Addis Ababa highlight the need for greater focus on the city’s women and children living in the poorest households and districts in maternal, newborn, and child health programs.

**Supplementary Information:**

The online version contains supplementary material available at 10.1007/s11524-024-00836-0.

## Introduction 

The “leave no one behind” principle underlying the 2030 Agenda for Sustainable Development underscores the need for equitable progress toward all Sustainable Development Goals (SDGs)—including Goal 3, which covers maternal, newborn, and child health (MNCH), and Goal 11, which calls for making cities safe, resilient, and sustainable. Much attention has been given to closing the gaps between rural and urban populations, but the considerable inequities within cities have been neglected, including the lack of basic services in many cities’ poorest areas. Intra-urban gaps are a major concern because the urban share of the total population is expected to rise to 60% over the next decade. Sub-Saharan Africa (SSA) has a high annual rate of urbanization at 3.9%, and by 2030, about 237 million people will living in urban poor areas [1].

According to UN-Habitat 2020 estimates, Addis Ababa, the capital and biggest city in Ethiopia, had an estimated population of over 5 million, with an additional estimated 2.7 million people living in surrounding places that can be considered part of the greater urban area [2]. The current urbanization level in Ethiopia stands at 21.1% [3] and is far lower than both the world average (55.7%) and Africa overall (40%) [4]. However, according to figures from 2015, the country has a high (5%) annual urbanization rate that is expected to continue indefinitely [5, 6].

Addis Ababa is a thriving city that attracts businesses and provides employment opportunities. As a result, there has been an increase in squatter settlements and urban slum areas, whose residents lack easy and consistent access to basic services such as healthcare [7]. Previous studies indicate that there are significant intra-urban inequalities even though access and utilization of reproductive, maternal, newborn, and child health (RMNCH) services are typically higher in urban areas than in rural areas across SSA [8, 9]. Skilled care during pregnancy, childbirth, and the postpartum period is critical in reducing maternal and neonatal morbidity and mortality.

According to the 2019 Ethiopia Development and Health Survey (EDHS), in Ethiopia, overall 43% of women received at least four antenatal care visits (ANC4), which is the global basic standard of care; 50% of births were attended by a skilled provider (SBA), defined as births attended by health professionals accredited by the Ministry of Health (MOH); 5% of the deliveries were conducted by Caesarian section (C-section), defined as deliveries conducted via surgical procedures; and 34% of women received postnatal care (PNC), defined as care provided in the first 2 days.

Addis Ababa residents have much better maternal and child health service utilization compared to their rural counterparts. Accordingly, 96% of women had SBA coverage, 24.1% of women delivered by C-section, and 74% of women received PNC in the first 2 days. However, significant maternal health service utilization disparities exist between the highest wealth quintile and the lowest quintile. Findings from the 2019 EDHS show that births to mothers in the highest wealth quintile (87%) are almost four times more likely to be assisted by a skilled provider than births to mothers in the lowest quintile (22%) [10].

As a study in Addis Ababa evidenced, both poor and non-poor attend ANC services from public health facilities. Meanwhile, 15.3% and 41.7% of poor and non-poor gave birth at private facilities, respectively [11]. Factors like educational status and household wealth status are associated with the preferred place of delivery in Addis Ababa [8, 9]. In line with this, only 50.3% of the mothers in slum areas initiated antenatal care service at the early stage, and 20.2% received adequate antenatal care [8, 10].

Although there are lots of investments in the immunization program, preventable diseases remain a major health problem among children in developing countries, including Ethiopia [10, 12]. Child immunization in Addis Ababa has remained below full coverage; for example, coverage of the third dose of the pentavalent vaccine (Penta3) was 93% in 2019. Moreover, the share of eligible children in Addis Ababa who had received all their recommended vaccines fell from 93% in 2016 to 83% in 2019.

The population groups most often under-immunized are those living in slum areas, illegal squatter settlements, and newly expanding semi-urban zones [12, 13]. To better understand why utilization remains below the expected level, further investigation that considers health facility factors and individual and community factors is needed.

There is limited research that explores the MNCH service utilization coverage disparity in urban poor and non-poor districts of Addis Ababa. This study specifically aims to examine temporal trends and spatial variations of MNCH service coverage and child mortality among the poor (bottom 40%) and non-poor (top 60%) districts and households in Addis Ababa city.

## Methods

### Study Area and Data Sources

This study was carried out in Addis Ababa, which is administratively divided into 10 sub-cities that altogether have 116 districts. Data for all districts were electronically accessible. In contrast to many other African cities, relatively wealthy and impoverished households in Addis Ababa often live in the same sub-cities, and there are no clearly defined poorest and richest districts across the overall urban area.

To identify poor districts within the city, we used a map of Addis Ababa city with administrative boundaries (sub-cities and districts), from the Ethiopian Mapping Agency (EMA), and the district-level poverty rate computed for Addis Ababa in 2017 by the World Bank using four dimensions of poverty including economic, social, environmental, and governance conditions with income and non-income poverty dimensions, whereas the top 60% (non-poor) and bottom 40% (poor) of households were determined using the household wealth index from five rounds of the EDHS.

According to the Addis Ababa Health Bureau, in 2021, there were 1133 health facilities in Addis Ababa city specifically, of which 988 (87%) were private clinics, 98 (9%) were public health centers, 43 (4%) were hospitals (12 public and 31 private), and less than 1% were facilities operated by nongovernmental organizations (NGOs) (three health centers and one clinic). Most private clinics provide other medical and specialized healthcare services, including dental, ophthalmic, dermatology, cardiac, and internal medicine. Data from and about private clinics are difficult to obtain. However, it is assumed that they provide only a relatively small share of MNCH services. Because private facilities are unaffordable to many people and their families and maternal health services are provided for free in public facilities in Ethiopia, most women turn to the public sector for maternal and child health services.

Health facility data from the District Health Information System 2 (DHIS2), collected and organized by the MOH, were used to identify the relative location of health facilities in all districts and to estimate trends in maternal health service coverage indicators. For this analysis, only full-year reports from public facilities (both hospitals and health centers), private facilities (hospitals and clinics), and NGO facilities (clinics and health centers) for 2019 to 2021 were used. Data extracted from five EDHS rounds, which were conducted from 2000 to 2019, were used to estimate trends of maternal health service utilization and child mortality.

### Data Analysis

We first located Addis Ababa city’s geographic boundaries of sub-city and districts using the administrative boundaries map. Then, the districts were classified into the bottom 40% (poor) and top 60% (non-poor) using the World Bank poverty rate index and positioned on the map of the city. Geographic coordinates for each facility were combined with the city map to identify the district in which each health facility is located. Therefore, we were able to assign each health facility to their corresponding bottom 40% or top 60% of districts.

To assess coverage, trends, and identify inequalities in access and utilization of MNCH services, we selected key MNCH indicators having consistent reporting in DHIS2 over 3 years (2019–2021) and five EDHS rounds (2000–2019). These were as follows: ANC4, iron and folic acid (IFA) supplementation, SBA, PNC, C-section delivery, post-abortion care (PAC) utilization, safe abortion care (SAC), child immunization antigen utilization such as Bacillus Calmette–Guérin (BCG) vaccine, third dose of pentavalent vaccine (Penta3), and measles-containing vaccine dose one (MCV1).

We also computed levels and trends of neonatal and under-5 mortalities (NMR and U5MR) per 1000 live births for the bottom 40% (poor) and the top 60% (non-poor) of households in Addis Ababa. Additionally, we utilized EDHS data to assess maternal health service utilization, harmonizing it with routine health facility data. To estimate maternal health service coverage between 2000 and 2019, we derived poverty status from all five rounds of EDHS surveys. The wealth index scores, based on dwelling materials, access to utilities, and household assets in urban areas, were generated through principal component analysis by EDHS. We then broke down the analysis of coverage indicators into two groups: the less affluent 40% and the more affluent 60% of households, focusing on a wealth index score recalibrated for urban Addis Ababa. This 40% threshold corresponds closely with the proportion of the population living below the UN-Habitat standard.

Our analysis concentrated on EDHS data from five surveys conducted from 2000 to 2019 within the urban cluster of Addis Ababa, encompassing all births over the previous 5 years. We evaluated maternal health intervention coverage indicators, which included the following: ANC4, IFA during pregnancy, SBA, C-section deliveries conducted via surgical procedures, and PNC. Point estimates of NMR and U5MR, with each data point representing a 10-year period preceding each of five rounds of EDHS surveys, were computed to explore the levels and trends among the wealthiest (top 60%) and poorest (bottom 40%) households The 10 years were used to ensure sufficient sample size for the analysis. A relative difference described as the absolute difference of mortality rates between EDHS: 2000 and 2019 relative to mortality 2000—and average annual rates of reduction of mortality rates were utilized further to describe inequalities observed in the pace of reduction among the wealthiest and poorest households during the two decades.

The identification of appropriate sources for district denominator populations, such as live births and children eligible for immunization, suitable to be used in combination with DHIS2 data was challenging because reliable up-to-date estimates are not available [14]. Population projections based on the latest population and housing census (2007) are the main source of sub-national population estimates in Ethiopia, but these lack the required precision and result in coverage estimates above 100%. We used an alternative approach to obtain denominators [15, 16]. Since at least one antenatal care visit (ANC1) and one dose of pentavalent vaccine (Penta1) population coverage were near-universal in Addis Ababa (more than 96% in the EDHS 2019) [10], the total numbers of ANC1 visits and Penta1 vaccinations in the reported facility data should be a good approximation of the target population. We added 3% to these numbers to include non-users and obtained estimates of all pregnant women and children eligible for immunization [16].

Temporal trend and spatial analyses were conducted to assess disparities in MNCH service coverage by district poverty status. The trend in service coverage was estimated annually and as an average over the years for which the data were available. Data were cleaned, and summary measures were analyzed using Stata version 14 software. Additionally, a one-tailed *t*-test was used to compare the mean coverage of MNCH services of the two groups. The geospatial analysis was conducted using QGIS software version 3.16, and maps were produced in ArcGIS pro version 2.5. To declare a statistically significant difference in the level of trend and service coverage and child mortality between the bottom 40% and the top 60% of districts and households, *P*-values and confidence levels were calculated. A *P*-value less than 0.05 was declared significant.

## Results

**Fig. 1:**
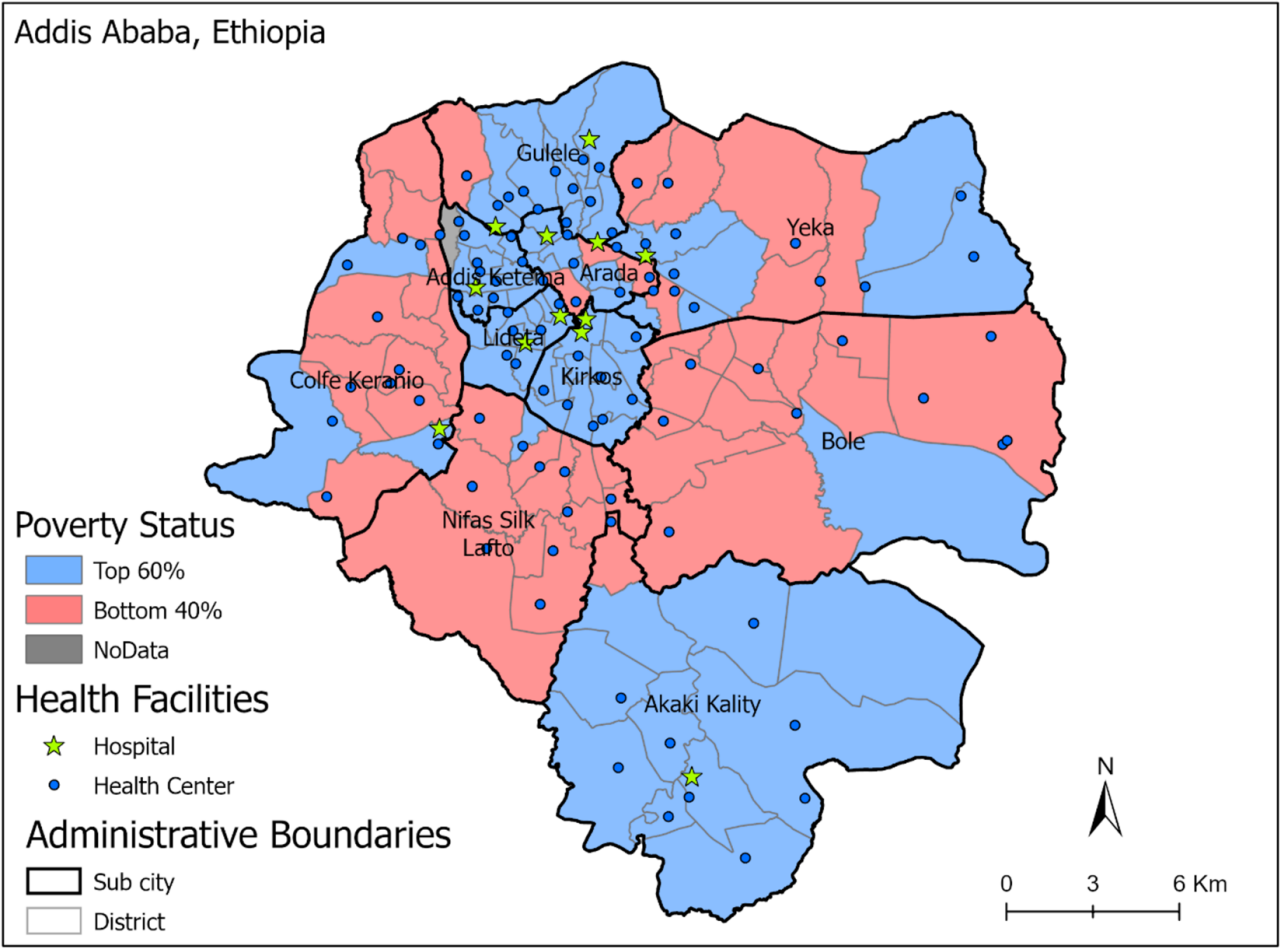
Map showing the poverty status of districts within Addis Ababa city and the relative location of public health facilities. Note: the 116 districts are within ten sub-cities

Figure 1 shows the spatial distribution of the poorest and richest districts according to our classification. The top (non-poor) 60% of districts (marked in blue) are predominantly located in the city center, and the outskirts with the bottom (poor) 40% of districts are located in between (in pink). Most public health centers and all public hospitals are located in the top 60% of districts. Private facilities are not displayed on the map.

### Health Facility Distribution Across the Bottom 40% and Top 60% of Districts

Out of a total of 1133 health facilities in Addis Ababa, 384 (33.9%) health facilities reported at least once via DHIS2 between 2019 and 2021. Of these, only 133 (35%) health facilities (all 110 public and 23 private) were geo-located and used for the analysis. A total of 79 (59.4%) health facilities (42.9% health centers, 15.8% hospitals, and 0.8% specialty centers) are located in the top 60% of districts, while 54 (40.6%) health facilities (27.1% health centers, 8.3% hospitals, 4.5% clinics, and 0.8% specialty center) are found in the bottom 40% of districts.

### Maternal and Child Health Services Coverage

Major differences in coverage were observed for all maternal health service coverage indicators. While coverage estimates in the top 60% of districts approximated universality, and in the case of C-section far exceeded population need, low coverage in the bottom 40% of districts is most notable for SBA (54%) and also ANC4 (67%). On the other hand, the C-section rate of 9% suggests that the need for C-sections (between 10 and 15% of births) is almost met in the bottom 40% of districts. On the other hand, both poor and non-poor districts are performing well in child immunization services. There is no significant difference between the bottom 40% and the top 60% in child vaccination coverage (Table 1).

**Table 1:** Aggregate maternal and child health services coverage in the poorest (bottom 40%) and wealthiest (top 60%) districts of Addis Ababa by poverty status, 2019–2021

Maternal and child service coverage	Poverty status		*P*-value*
	Bottom 40%	Top 60%	
ANC4	67.0%	91.7%	0.00
IFA supplementation	84.0%	88.9%	0.30
SBA	54.3%	100%	0.00
C-section	9.1%	33.6%	0.00
PNC	62.3%	96.9%	0.00
PAC	3.2%	6.6%	0.19
SAC	10.6%	6.6%	0.32
BCG	94.0%	100%	0.08
Penta 3	93.0%	95%	0.07
MCV1	91.0%	91%	0.09

*P-value, significant at < 0.05. *ANC4* at least four antenatal care visits, *IFA* iron and folic acid, *SBA* skilled birth attendance, *C-section* cesarean section, *PNC* postnatal care within 2 days after birth, *PAC* post-abortion care, *SAC* safe abortion care, *BCG* Bacillus Calmette–Guérin vaccine, *Penta 3* third dose of pentavalent vaccine, *MCV 1* measle 1st dose vaccination (note: numbers do not add up to 100% as they represent different districts and services)

**Fig. 2:**
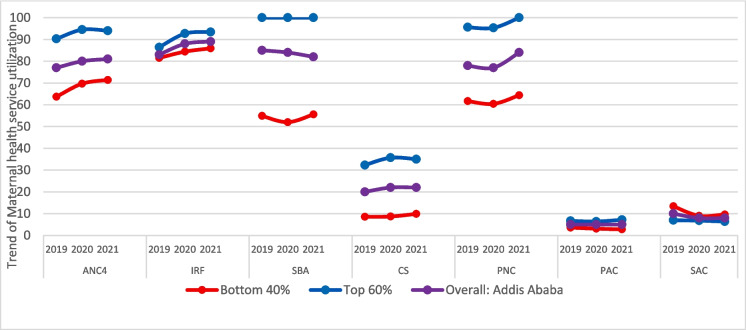
Trends of maternal health service coverage in urban Addis Ababa, 2019–2021. Note: ANC4, at least four antenatal care visits; IFA, iron and folic acid supplementation; SBA, skilled birth attendance; C-section, cesarean section; PNC, postnatal care within 2 days after birth; PAC, post-abortion care; SAC, safe abortion care

Figure 2 presents trends of maternal health service coverage over 3 years among the non-poor (top 60%) and poor (bottom 40%) districts. ANC4 service coverage during 2019 to 2021 was higher in the top 60% of districts than in the bottom 40% of districts. Between 2019 and 2020, the annual rate of change in ANC4 coverage was 4.6%, but this decreased to − 0.4% in the following year. However, the overall average change in ANC4 coverage was 2.1%, which was not statistically significant.

In districts in the bottom 40%, ANC4 coverage increased by 9.4% annually between 2019 and 2020 and by 2.4% annually between 2020 and 2021, for an average change of 5.9%. This suggests that ANC4 coverage is trending upward in both the top 60% and bottom 40% of districts, with a slightly higher rate of increase in the latter.

Skilled birth attendance in the top 60% (non-poor) of districts was 100% in 2019, 2020, and 2021. This implies that maximum coverage was achieved in the top 60% of districts. The situation was substantially worse in the bottom 40% (poor) of districts, where SBA coverage remained below 60% during 2019 to 2021. In terms of SBA, the annual change in the bottom 40% of districts from 2019 to 2020 was − 5.2%, and from 2020 to 2021, it was 6.9%. The SBA’s average change between 2019 and 2021 was 0.8%, indicating a modest upward trend.

PNC service coverage was also higher near 96.9% in the non-poor districts, while it remained below 63% in the bottom 40% (poor) of districts during 2019–2021. Overall, maternal health services coverage was considerably higher in the top 60% of districts than in the bottom 40%. Between 2019 and 2020, postnatal care utilization coverage in the top 60% of districts decreased by 0.3% annually. However, it increased by 7.5% annually from 2020 to 2021, for an average change of 3.6% over the 2-year period.

In the bottom 40% of districts, postnatal care utilization coverage decreased by 2.1% annually from 2019 to 2020 and increased by 6.6% annually from 2020 to 2021, for an average change of 2.3% over the 2-year period. Overall, maternal health service coverage remained higher in the wealthiest (top 60%) than poorest (bottom 40%) districts (Supplement Fig. 1 and Table 1).

In addition to using DHIS2, we analyzed five rounds of the Ethiopia Demographic and Health Survey (EDHS) from 2000 to 2019 to understand the disparity in coverage of key maternal health interventions between poorer households (bottom 40%) and richer households (top 60%).

**Fig. 3:**
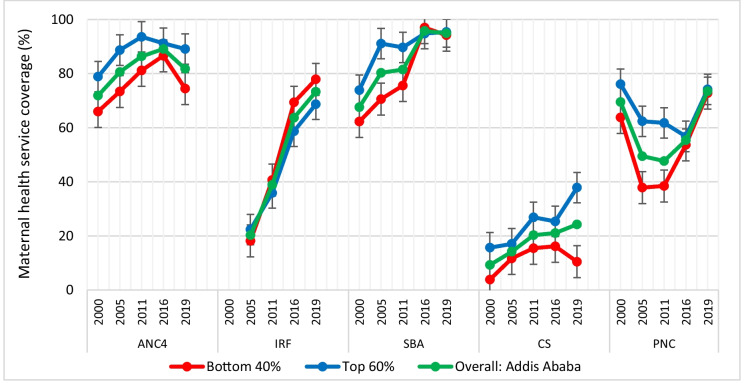
Trends of maternal health service coverage for ANC4, IFA, SBA, C-section*,* and PNC comparing the bottom 40% and top 60% in Addis Ababa, EDHS 2000*–*2019*.* Note: *ANC4*, at least four antenatal care visits; IFA, iron and folic acid supplement; SBA, skilled birth attendance; C-section, cesarean section; PNC, postnatal care

Figure 3 shows that ANC4 coverage has consistently been higher for pregnant women in richer households than among those in poorer households in Addis Ababa over the past 20 years.

IFA supplementation has been higher among women in poorer households than women in richer households, except in 2005. SBA coverage was higher among urban richer women than urban poorer women in the EDHS: 2000, 2006, and 2011. Notably, although SBA coverage has been lower among women in poorer households, there was a larger increase among this group from the 2011 EDHS (75.6%) to the 2016 EDHS (97.0%). The gap between the richer and poorer groups closed between EDHS-2016 and EDHS 2019.

Coverage for C-section was lower among women in the bottom 40% than among women in the top 60% across all five EDHS periods (2000 to 2019). Rates started low, at around 3.9% in the bottom 40% in 2000, before increasing to 11.7% in 2005 and beyond. The rate of C-sections is higher than the recommended WHO level among women in the top 60%, while remaining within the range of meeting the need (between 10 and 15%) among women in the bottom 40% between 2005 and 2019.

Postnatal care within 2 days has exhibited an inconsistent trend both among women in the bottom 40% and women in the top 60% over the past 20 years. Generally, PNC coverage is higher among women in the top 60% across all five EDHS periods.

**Fig. 4:**
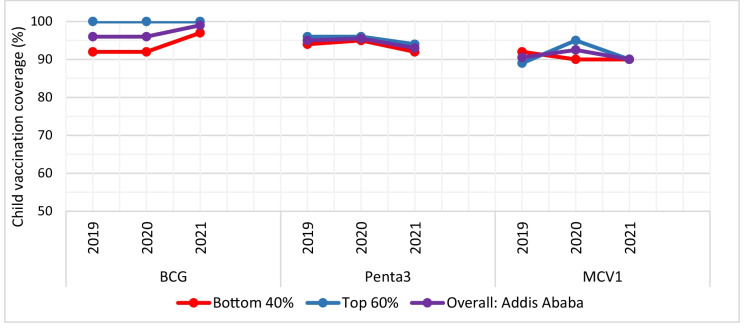
Trends of child vaccination coverage in urban Addis Ababa, 2019–2021. Note: BCG, Bacillus Calmette–Guérin vaccine; Penta 3, third dose of pentavalent vaccine; MCV1, measles-containing vaccine dose one

As shown in Fig. 4, BCG vaccination coverage in the bottom 40% of districts was 92% in 2019 and 2020 and then increased to 97% in 2021. Penta3 vaccination coverage increased from 94% in 2019 to 95% in 2020 and declined back to 94% in 2021. From 2019 to 2021, the BCG vaccination rate in the top 60% of districts stays 100%. The Penta3 vaccine shows a declining trend, with an average change of − 1.0% between 2019 and 2021 and an annual change rate of − 2.1% between 2020 and 2021.

Measles vaccination coverage decreased from 92% in 2019 to 90% in 2020 and remained at that level in 2021. Conversely, between 2019 and 2020, MCV1 grew at an annual change rate of 6.7%. Subsequently, there was a decline with an average change rate of 0.7% and an annual change rate of − 5.3% between 2020 and 2021 (*P*-value above 0.05).

Within the districts that comprise the lowest 40%, the coverage of BCG vaccination was 92.0% in 2019 and 2020, but it rose to 97.0% in 2021, signifying a 5.4% increase and an average shift of 2.7% from 2019 to 2021. The annual rate of change in Penta3 vaccine coverage was 1.1%, − 3.2% between 2019 and 2020, and 2020 and 2021, respectively. Between 2019 and 2021, there was a − 1.1% average change in Penta3. There has been a decline in the measles vaccine coverage between 2019 and 2020, with an annual change rate of − 2.2% and an average change of − 1.1%, suggesting a downward trend.

Overall, high vaccination rates are found in both the top 60% and bottom 40% of districts, indicating a narrowing of the immunization service gap between poor and non-poor districts. BCG coverage is generally trending upward in both districts (Fig. 4).

### Spatial Distribution of Maternal and Child Health Service Coverage

**Fig. 5:**
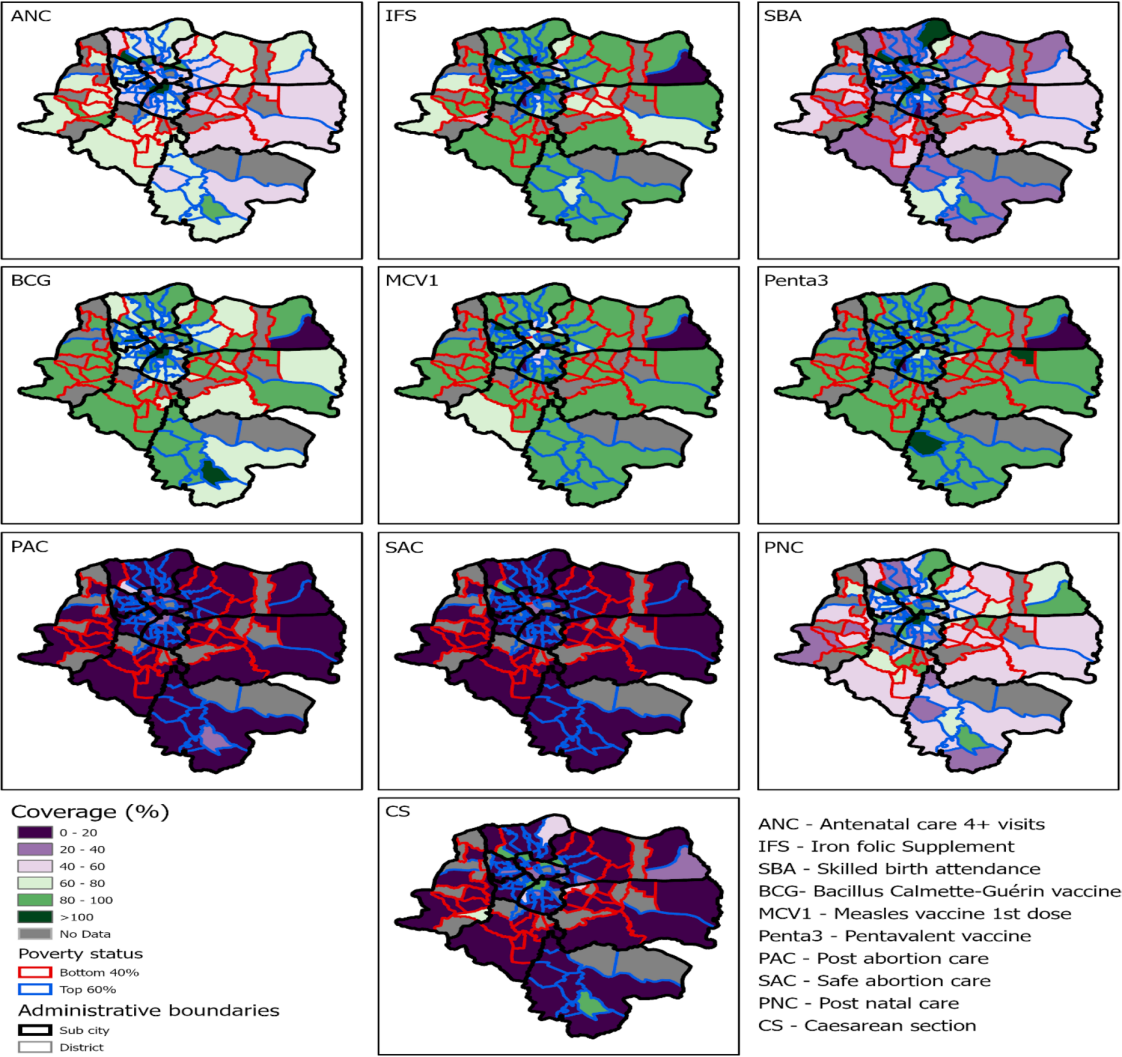
Geo-spatial distribution of MNCH services coverage at the sub-city level, 2019–2021

Figure 5 below presents maternal and child health service coverage by district. The majority of the top 60% of districts have ANC4 coverage exceeding 60%, while coverage of ANC4 within the bottom 40% of districts falls in the range of 20–60%. The average of both the top 60% and bottom 40% of districts have high (> 80%) IFA supplementation coverage.

Regarding SBA and PNC, an average of the top 60% of districts have higher coverage compared to the bottom 40% of districts. C-sections, which can be lifesaving interventions for mothers at risk, are higher in the top 60% of districts than the bottom 40% ones. However, while there is overuse of C-section services in some of the top 60% of districts, the service is underused in the bottom 40%.

In contrast to the distinct differences over several indicators of maternal health service, both the top 60% and bottom 40% of districts have high child immunization coverage greater than 80% BCG, Penta3, and MCV1 coverage in both classifications (Fig. 5).

### Newborn and Under-5 Mortality

**Fig. 6:**
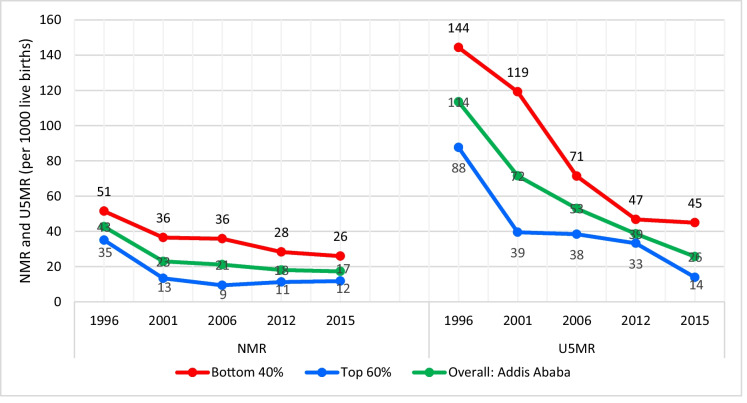
Trends of NMR and U5MR in urban Addis Ababa, 1996–2015. Note: Data source: five rounds of EDHS surveys: 2000–2019, and each data point represents 10-year period preceding each EDHS: 2000–2019, and the year represents the midpoint within the 10-year period preceding each survey. NMR, neonatal mortality rate; U5MR, under-5 mortality rate

Figure 6 illustrates the levels and trends of NMR and U5MR per 1000 live births for urban Addis Ababa across the two decades from 1996 to 2015 (each data point represents the 10-year period preceding each EDHS: 2000, 2005, 2011, 2016, and 2019). These rates are analyzed for the overall population, as well as specifically for the poorest (bottom 40%) and the wealthiest (top 60%) households in urban Addis Ababa. These findings reveal that both NMR and U5MR exhibited substantial declines, with NMR decreasing by 65% and U5MR by 77% over the two decades. This translates to an average annual reduction of 5% for NMR and 8% for U5MR. However, the results diverge when considering household wealth status.

The decline in U5MR is characterized by a significant drop among the poorest (bottom 40%) households, declining from 144 to 45 deaths per 1000 live births, marking a 69% reduction at an average annual rate of 6%. In contrast, U5MR among the wealthiest (top 60%) households decreased from 88 to 14 deaths per 1000 live births, reflecting a 84% reduction at an average annual rate of 10% between EDHS 2000 and EDHS 2019.

Similarly, NMR experienced a 49% decline among the poorest (bottom 40%) households, decreasing from 51 to 26 deaths per 1000 live births, with an average annual drop of 4%. Among the richest (top 60%) households, NMR declined by 66%, reducing from 35 to 12 deaths per 1000 live births, at an average annual rate of 6%, during the same time frame.

## Discussion

MNCH services in Addis Ababa are primarily delivered through a network of primary healthcare units, with the majority of these being public health centers. For cases that are complex and require advanced care, patients are referred to hospitals, which also serve mothers and children not only from districts within Addis Ababa but also from other parts of the country. The significant patient load in many hospitals contributes to higher coverage of basic maternal and child health services in their respective districts.

Based on the data available, this study demonstrated that 59.4% of public health facilities, particularly most public hospital that provide free or subsidized services, are situated within the districts representing the non-poor (top 60%) districts [11]. In the realm of private healthcare providers, only a limited number of private clinics offer maternal and child health services. Consequently, their impact on key maternal and child health indicators remains relatively modest. It is worth noting that approximately one-third of all health facilities (and roughly a quarter of private facilities) report data to the District Health Information System (DHIS2).

In the past two decades, there have been significant advancements in the coverage of key maternal health services in Addis Ababa, including ANC4, IFA supplementation, SBA, C-section, and PNC coverage for women in Addis Ababa. Notably, improvements in ANC4 and SBA have been more pronounced among households within the bottom 40% of the socioeconomic spectrum, effectively reducing disparities, by the top 60% of households.

In general, the coverage of MNH services is higher among the wealthiest (top 60%) than the poorest (bottom 40%) districts (referring to DHIS2 data) and households (referring to EDHS data). This finding aligns with prior research, which suggests that women in economically disadvantaged communities often face challenges in accessing maternal services, even when these services are geographically close to well-functioning facilities [17]. One potential explanation for these persistent disparities is that women residing in economically disadvantaged areas may lack access to essential services because they live in informal settlements or areas with limited medical infrastructure. In line with this, Addis Ababa is characterized by rapid urbanization, exhibits a complex and evolving settlement pattern. The city center hosts government offices, businesses, and affluent residents, while the outskirts are inhabited by working-class individuals and immigrants. The city also experiences the rapid growth of informal settlements, marked by substandard housing, inadequate basic services, and overcrowding, all of which impact health service delivery. This settlement pattern has numerous implications for the city’s development, leading to wealth concentration, inequality, and social exclusion, particularly in the city center [18].

The study’s findings, which indicate nearly universal maternal health service access in the top 60% (non-poor) of districts, suggest more successful health-seeking behavior and the presence of highly concentrated health facilities in these areas, contributing to improved coverage.

The study also reveals C-section rates exceeding 10% in the poorer 40% districts and over 15% in the richer 60% districts. Rates of SBA are relatively similar, exceeding 60% in the poorer 40% districts and over 70% in the richer 60% districts, respectively.

Generally, living in the poorest (bottom 40%) districts is consistently linked to lower utilization of ANC4, SBA, C-section, and PNC. The disparities in coverage of ANC4, SBA, C-section, PNC, and PAC services between the richest and poorest districts in Addis Ababa are substantial, with absolute difference of 24.7%, 45.7%, 24.5%, 34.6%, and two-fold, respectively.

ANC4 coverage has been on the rise in both district contexts, with slightly more pronounced improvements observed in the poorest districts. Similar to findings from a previous study conducted in Addis Ababa [11], the disparity in ANC4 coverage among women residing in the wealthiest and poorest districts may be attributed to the challenges faced by working women from economically disadvantaged areas. These challenges include labor-intensive, low-paying jobs that leave them with limited time to visit healthcare facilities, as well as financial constraints related to transportation and expensive diagnostic services in referral facilities.

The aggressive strategy of the MOH to supplement all pregnant women with IFA during the first ANC checkup and throughout pregnancy in order to reduce the risk of developing anemia and related complications may be responsible for the high coverage among pregnant women in both the top 60% and bottom 40% of districts. Many women in the bottom 40% of districts in particular may have low hemoglobin related to nutritional status; therefore, some facilities provide IFA based on hemoglobin levels, an intervention that could independently increase the coverage. The higher availability of SBA services in richer districts, characterized by a greater number of public health facilities (referring to hospitals and health centers), and specialized maternal and child clinics, combined with the increased economic empowerment and decision-making authority of women living in these areas, contributes to the nearly two-fold difference in SBA rates between the wealthiest and poorest districts. Another contributing factor is the limited expansion of maternity waiting homes in public health facilities throughout urban Ethiopia, including Addis Ababa. These homes were designed to enhance SBA utilization among economically disadvantaged women but have not been widely extended yet [19].

Based on DHIS2 data from 2019 to 2021, there is a substantial contrast in C-section rates between different socioeconomic districts. Nearly one-third (33.6%) of mothers in the wealthiest districts received C-section deliveries, while in the poorest (bottom 40%) districts, only 9.1% underwent C-sections This C-section rate in the wealthiest (top 60%) districts exceeds the WHO’s recommended population-level C-section rate of 10–15% and is notably higher than the overall rate across all of Addis Ababa (24.1%) as reported in another study [10]. The relatively elevated C-section levels in the wealthiest districts may be linked to the high SBA coverage, of whom many of them may opt for non-elective C-sections in private healthcare facilities, despite the associated costs, in an attempt to avoid birth-related pain.

The significant difference in PNC coverage between poorer (bottom 40%) districts, standing at 62.3%, and non-poor (top 60%) districts with a near-universal coverage of 96.9% may be attributed to the greater challenges faced by economically disadvantaged women in accessing care, a phenomenon observed in a study conducted in a city in North India [20]. Additionally, the substandard quality of services provided by many lower-level healthcare facilities in the bottom 40% of districts in Addis Ababa, as documented in another study among impoverished urban neighborhoods in Kenya [19, 20], could be contributing to this disparity. Furthermore, the inability of many economically disadvantaged urban women to cover transportation costs, as observed in other research [21], may also be a factor. Notably, there are no significant disparities or trends in PAC and SAC.

The data sets utilized in this study consistently indicate that maternal health service coverage is higher in the non-poor (top 60%) districts and households, implying that DHIS2 can effectively be used for estimating district-level service coverage, as its results align with that of the EDHS.

The child immunization coverage gap between the bottom 40% and the top 60% of districts is narrowing and both districts have high child vaccination coverage due to the expansion of the urban health extension program, which is supported by frequent immunization campaigns in urban areas [22]. However, although BCG coverage has a positive trend, both Penta3 and MCV1 revealed either a declining or stalling trend. These declining vaccination trends in the bottom 40% of districts could be related to difficulties in access, including service delivery disruptions associated with the COVID-19 pandemic that affected many poorer women during 2019 to 2021. One possible factor behind BCG coverage improvements in the top 60% of districts could be increased SBA and early PNC service utilization. The declining trend of Penta3 vaccination in the top 60% of districts might be attributed to the failure to complete child vaccination regimens due to forgetting subsequent doses, as well as negative experiences associated with vaccine side effects. The rates of neonatal and under-5 mortalities were consistently higher in the bottom 40% of households compared to the top 60%. Recent trends indicate that NMR has remained stagnant in wealthier areas but decelerated in poorer ones. In the case of U5MR, there has been a decline in poorer households and stagnation in wealthier ones. This shift may be attributed to the diminishing urban advantage over rural areas in terms of various health, social, and economic factors, particularly in rapidly growing cities with expanding informal settlements.

Failure to adequately address the needs of the growing urban poor, improve their living conditions, and enhance their health status could impede progress in underdeveloped areas, consequently affecting the overall reduction of child mortality. Notably, between 2000 and 2005, NMR and U5MR decreased more rapidly among the top 60% of households compared to the bottom 40% of households. However, the rate of reduction slowed for both groups between EDHS-2005 and EDHS-2011.

A concerning observation is the stagnation in NMR among the top 60% of households between 2011 and 2016, while under-5 mortality rates decreased significantly during the same period. Both groups experienced decreases in NMR and U5MR between 2016 and 2019. Nevertheless, the average rate of change in under-5 mortality rates across the two decades was higher in the top 60% of households than in the bottom 40%.

This study has several limitations to consider. These include incomplete data and a lack of linkage to a well-defined target population within DHIS2. Some private facilities provided inconsistent and incomplete reporting, leading to their exclusion from the analysis. The study also faced challenges in precisely identifying urban slum areas in Addis Ababa, and the DHIS2 data set lacks household-level information required to create distinct clusters for analysis. Despite these limitations, the combined strengths of both datasets offer valuable insights. While DHIS2 lacks individual-level data for equity analysis, it does provide information on service availability and volume. In contrast, EDHS data offers individual and household characteristics of service users for equity analysis but lacks details on service availability and volume.

This study represents the first attempt to use DHIS2 for assessing the coverage of maternal, newborn, and child health services at a lower administrative level in the Ethiopian context. By integrating these datasets with population estimates, health facility locations, and their characteristics, this approach provides previously unavailable insights into service coverage and equity in Addis Ababa. This method holds the potential to be valuable for future research in cities within the region.

## Conclusion

Maternal health service coverage, except for abortion care, is significantly lower in economically disadvantaged districts compared to their more affluent counterparts in Addis Ababa. This difference is less pronounced in childhood vaccination coverage. However, it is important to note that MR and U5MR were higher in the city’s most impoverished households.

In summary, Addis Ababa exhibits substantial inequalities in MNCH service utilization as well as neonatal mortality. These disparities underscore the pressing need for a heightened focus on improving the health of women and children living in the most economically challenged conditions. To address these disparities effectively, it is advisable for the MOH to establish a link between the DHIS2 system and the catchment area population while also incorporating all private and NGO health facilities into the DHIS2 reporting system.

## Supplementary Information

Below is the link to the electronic supplementary material.

Supplementary file1 (DOCX 138 KB)

## Data Availability

Data for the study are available upon reasonable request from Ethiopian Ministry of health (email: moh@ethionet.et)
