# Supplementary material for: Inter-district and Wealth-related Inequalities in Maternal and Child Health Service Coverage and Child Mortality within Addis Ababa City
Source: J Urban Health. 2024 Mar 27;101(Suppl 1):68–80. doi: 10.1007/s11524-024-00836-0 (PMC11602917; doi:10.1007/s11524-024-00836-0)
Supplement: Supplementary file 1 — Supplementary file1 (DOCX 138 KB) [file 11524_2024_836_MOESM1_ESM.docx]

**Supplementary materials**

**Supplementary Figure 1**. Levels and trend of maternal health service coverage from EDHS: 2000-2019 and DHIS2: 2019-2021


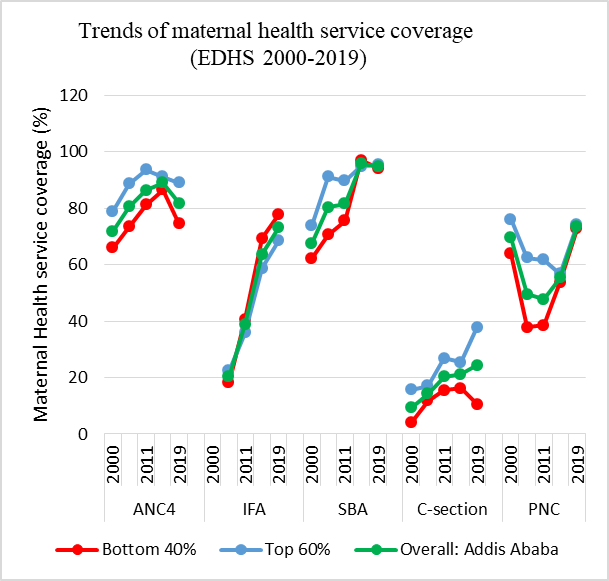

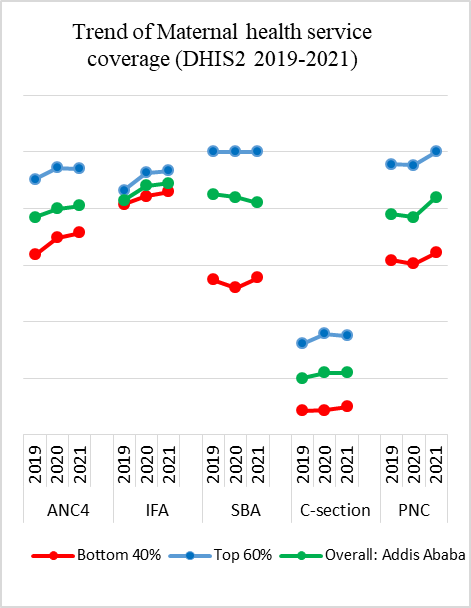


| **Supplementary Table 1.** Maternal health service coverage among the poorest (bottom 40%) and wealthiest (top 60%) households and 95%CI, data from five rounds of EDHS: 2000–2019 | | | | | | | |
| --- | --- | --- | --- | --- | --- | --- | --- |
| variable | EDHS years | Poorest (bottom 40%) households | | | Wealthiest (top 60%) households | | |
|  |  | Estimate | LB | UB | Estimate | LB | UB |
| ANC4 | 2000 | 66.0 | 57.2 | 74.8 | 78.9 | 71.8 | 86.2 |
|  | 2005 | 73.4 | 66.1 | 80.7 | 88.7 | 83.4 | 94.1 |
|  | 2011 | 81.2 | 74.7 | 87.7 | 93.6 | 89.9 | 97.3 |
|  | 2016 | 86.6 | 80.5 | 92.7 | 91.2 | 87.3 | 95.1 |
|  | 2019 | 74.5 | 68.3 | 80.8 | 89.1 | 84.7 | 93.5 |
| IFA | 2000 | N/A | N/A | N/A | N/A | N/A | N/A |
|  | 2005 | 18.2 | 11.3 | 25.5 | 22.4 | 14.5 | 30.3 |
|  | 2011 | 40.7 | 32.5 | 48.9 | 35.9 | 27.7 | 44.1 |
|  | 2016 | 69.4 | 60.3 | 78.4 | 58.7 | 51.2 | 66.2 |
|  | 2019 | 77.9 | 71.8 | 84.1 | 68.7 | 57.1 | 80.3 |
| SBA | 2000 | 62.3 | 54.1 | 70.5 | 73.9 | 66.5 | 81.3 |
|  | 2005 | 70.6 | 60.9 | 80.3 | 91.1 | 85.9 | 96.3 |
|  | 2011 | 75.6 | 66.7 | 84.5 | 89.7 | 84.7 | 94.7 |
|  | 2016 | 97.0 | 94.6 | 99.4 | 94.8 | 91.8 | 97.9 |
|  | 2019 | 94.2 | 91.6 | 97.0 | 95.4 | 92.2 | 98.7 |
| C-section | 2000 | 3.9 | 1.4 | 6.3 | 15.7 | 10.6 | 20.7 |
|  | 2005 | 11.7 | 5.1 | 18.3 | 17.1 | 10.5 | 23.6 |
|  | 2011 | 15.5 | 9.7 | 21.2 | 26.9 | 17.5 | 36.2 |
|  | 2016 | 16.2 | 10.9 | 21.4 | 25.4 | 18.9 | 31.7 |
|  | 2019 | 10.5 | 4.2 | 16.8 | 37.9 | 29.2 | 46.7 |
| PNC | 2000 | 63.8 | 56.0 | 71.6 | 76.1 | 68.4 | 83.7 |
|  | 2005 | 37.9 | 28.1 | 47.9 | 62.4 | 53.4 | 71.4 |
|  | 2011 | 38.5 | 28.3 | 48.6 | 61.8 | 49.4 | 74.1 |
|  | 2016 | 53.7 | 40.4 | 67.1 | 56.8 | 45.9 | 67.6 |
|  | 2019 | 72.8 | 59.9 | 85.8 | 74.2 | 64.2 | 84.2 |

Note: ANC4: at least four antenatal care visits, IFA: iron and folic acid supplementation, SBA: skilled birth attendance, C-section: Cesarean section, PNC: postnatal care; LB = 95% lower band; UB = 95% upper band; N/A: data not available.
